# Short Bowel Syndrome and Kidney Transplantation: Challenges, Outcomes, and the Use of Teduglutide

**DOI:** 10.1155/2020/8819345

**Published:** 2020-10-06

**Authors:** Elizabeth Abou Diwan, Ankit B. Patel, Alex G. Cuenca, Nahel Elias, Hannah M. Gilligan, Eliot Heher, David E. Leaf, David Wojciechowski, Kassem Safa

**Affiliations:** ^1^American University of Beirut Medical School, Beirut, Lebanon; ^2^Division of Renal Medicine, Brigham and Women's Hospital, Boston, MA, USA; ^3^Division of Nephrology, Massachusetts General Hospital, Boston, MA, USA; ^4^Harvard Medical School, Boston, MA, USA; ^5^Transplant Center, Massachusetts General Hospital, Boston, MA, USA

## Abstract

Among patients with short bowel syndrome who commonly have kidney disease, kidney transplantation remains challenging. We describe the clinicopathologic course of a 59-year old man with short bowel syndrome secondary to Crohn's disease who underwent a deceased donor kidney transplant that was complicated by recurrent acute kidney allograft injury due to volume depletion from diarrhea, ultimately requiring the placement of permanent intravenous access for daily volume expansion at home resulting in the recovery of allograft function. Teduglutide treatment at 1.8 years post-transplant led to a dramatic decrease in diarrhea. A literature review of similar cases yielded 18 patients who underwent 19 kidney transplants. Despite high rates of complications, at the time of last follow-up (median 2.1 years [0.04-7]), 94% of the patients were still alive and 89% had functioning allografts, with a median eGFR of 37.5 [14-122] ml/min/1.73m^2^. In conclusion, despite high rates of complications, kidney transplantation in patients with short bowel syndrome is associated with acceptable short- and midterm outcomes. Further, we report for the first time the effects of the glucagon-like peptide-2 analogue teduglutide for short bowel syndrome in a kidney transplant recipient.

## 1. Introduction

Short bowel syndrome (SBS) is a cause of significant morbidity among patients with Crohn's disease, necrotizing enterocolitis, abdominal trauma, and other gastrointestinal conditions [1]. Among other complications, patients with SBS are at an increased risk for developing chronic- and end-stage kidney disease (ESKD). Further, patients with SBS are a high-risk population to manage following kidney transplantation, given recurrent bouts of volume depletion secondary to severe diarrhea, as well as enteric hyperoxaluria, which is a risk factor for allograft oxalate nephropathy [2]. There are only limited data on patient and allograft survival rates in this group of patients following kidney transplantation. In this report, we describe a patient suffering from Crohn's disease, who had SBS secondary to multiple small bowel resections, as well as ESKD of unclear etiology, who received a deceased donor kidney transplant, that was complicated by recurrent episodes of acute kidney injury (AKI) due to volume depletion from high diarrheal output. These episodes responded well to daily at-home intravenous fluid (IVFs) replacement, and he was subsequently treated with the glucagon-like peptide (GLP)-2 analogue teduglutide with improvement of the diarrhea. We also review all previously reported cases of kidney transplantation in the setting of SBS and summarize demographics, complications, and patient and allograft survival.

### 1.1. Case Presentation and Review of the Literature

We describe a 59-year old man with long-standing Crohn's disease complicated by subtotal colectomy and multiple small bowel resections leading to SBS, with only 150 cm of residual small bowel. As a result, he suffered from chronic diarrhea and developed chronic kidney disease (CKD), which progressed to ESKD over 7 years. He was on hemodialysis for 6 months before he underwent a deceased donor kidney transplant. The Kidney Donor Profile Index was 24%. Kidney biopsy obtained at the time of procurement had 1% glomerulosclerosis, 2% interstitial fibrosis, and acute tubular injury. He received induction therapy with basiliximab and maintenance therapy with mycophenolic acid, tacrolimus, and prednisone. He was discharged home on postoperative day 4 after requiring two sessions of hemodialysis for hyperkalemia.

For the next 8 weeks, his course was complicated by recurrent episodes of AKI secondary to volume depletion from diarrhea. His stool output exceeded 3.5 l per day at times. He presented with weight loss of 4.5 to 5 kg during each AKI episode, despite aggressive oral hydration. A kidney biopsy revealed acute tubular injury with rare tubular oxalate crystals. Infectious etiologies of diarrhea were ruled out. Despite transition from mycophenolic acid to azathioprine (to eliminate potential mycophenolate-induced gastrointestinal toxicity as a contributing factor), as well as aggressive motility inhibition including opium tincture, he continued to have high-output diarrhea and required four brief hospitalizations for volume expansion. On each occasion, volume expansion resulted in the resolution of the AKI. Eventually, he received an internal jugular venous Port-A-Cath and was discharged on a home regimen of 1 l of IVFs per day. This resulted in a sustained recovery of renal function up to 2.6 years after transplantation (at the time of this report), with steady serum creatinine of 0.9-1.1 mg/dl (eGFR 67 ml/min/1.73m^2^), stable body weight, and no additional hospitalizations since the third month posttransplant. Of note, over the last 9 months, he has had much less diarrhea and discontinued opium tincture after the initiation teduglutide (1.8 years after transplant). He has been tolerating the agent very well and was able to reduce home IVFs to every other day regimen with stable kidney allograft function. He maintains a low oxalate diet and takes calcium carbonate with meals. At eight months posttransplant, a 24-hour urine study showed a volume of 2.74 l, oxalate of 35 mg (reference range 20-40), calcium of 110 mg (reference range < 250), and calcium oxalate supersaturation of 2.47 (reference range 6-10). Despite requiring IVFs, the patient reported dramatic improvement in his quality of life posttransplant, with excellent functional status and full-time employment.

To identify all previously published cases of patients with SBS who underwent kidney transplantation, we conducted a *PubMed* search using the following keywords: short bowel syndrome; kidney transplantation; end-stage kidney disease; end-stage renal disease; enteric hyperoxaluria; and oxalate nephropathy. We identified nine reports of nineteen kidney transplants in 18 patients, published between 1988 and 2017 (one patient received two kidney transplants). We compiled relevant data on demographics, etiology of kidney disease, complications after transplantation, immunosuppressive regimens, long-term kidney allograft function, and patient and graft survival. Clinical characteristics of these patients are summarized in Tables 1 and 2. Patients were predominantly male (65%) and middle-aged (median age 53 years [18-74]) at the time of transplantation. Crohn's disease was the most common etiology of SBS, and the most prevalent cause of kidney disease was oxalate nephropathy. The median time from transplantation to last follow-up was 2.05 years [0.04-7]. Post-transplant complications were common, in particular delayed graft function, AKI, and recurrent oxalate deposition. All patients (94%), but one, were alive at the time of last reported follow-up, and among the 19 transplants, seventeen allografts (89%) were functioning, with a median eGFR of 37.5 ml/min/1.73m^2^ and a range of 14 to 122.

## 2. Discussion

SBS is a malabsorptive condition most often caused by a resection of greater than two thirds of the small bowel. In adults, SBS usually results from Crohn's disease, trauma, malignancy, radiation, mesenteric ischemia, or postoperative vascular and obstructive catastrophes [1]. SBS is associated with an increased risk of CKD due to a myriad of etiologies: chronic volume depletion from high-output diarrhea, inflammatory bowel disease (IBD) therapy-induced interstitial nephritis [3], and nephrolithiasis and nephrocalcinosis [2] due to low urine pH, low urine volume, and enteric hyperoxaluria [4].

The underlying pathophysiology of SBS predisposes patients who undergo kidney transplantation to a higher than usual complication rate. Volume depletion and AKI with acute tubular injury in the allograft were highly prevalent in the reported cases. Measures to reduce bowel motility and substitution of mycophenolic acid to azathioprine may not be sufficient, as was the case with our patient. In such cases, consistent and regular volume repletion may be needed to maintain adequate kidney function [5, 6]. Teduglutide is a GLP-2 analogue that increases intestinal absorption via enhanced epithelial cell growth and increased blood flow to the gut [7] and has been shown to decrease the need for parenteral support in patients with SBS [8]. To our knowledge, we report the first case of teduglutide use in a kidney transplant recipient. Our patient tolerated the drug well—and it allowed for the discontinuation of tincture of opium. Further, the frequency of home IV fluid administration was reduced in half, and the graft function remains excellent 9 months after starting the agent.

The most prevalent maintenance immunosuppression regimen in the reviewed cases was standard triple therapy with mycophenolate, tacrolimus, and prednisone. Of note, it is conceivable that patients with SBS will have medication malabsorption, yet in our patient there was no difficulty in achieving therapeutic tacrolimus levels (median tacrolimus level 5.6 ng/dl over the follow-up time); further, in the reviewed literature, the rejection rate reported was 15% (Table 2), suggesting that inadequate immunosuppression is not a limiting issue in this patient population. Indeed, shorter segments of small bowel are associated with higher tacrolimus bioavailability [9, 10] likely related to the partial metabolism of calcineurin inhibitors in the small bowel via intestinal CYP3A4 and the role of P-glycoprotein efflux of tacrolimus back into the intestinal lumen [11]. Further, calcineurin inhibitor-sparing strategies using costimulation blockade with belatacept infusions could be considered in select cases when therapeutic tacrolimus levels are difficult to achieve or to abate tacrolimus nephrotoxicity.

Kidney transplantation offers several advantages compared to dialysis in terms of survival, quality of life, and cost [12]. It is not clear whether patients with SBS enjoy similar advantages after kidney transplantation. Based on this report, it appears that kidney transplantation offers favorable outcomes for patients with SBS, and out of the nineteen reported patients, only one was deceased from shock and multiorgan failure early after transplant [5] (5%), while one other patient lost their allograft after 6 years requiring a second kidney transplant [2] (5%). Furthermore, these patients maintained a median eGFR of 37.5 ml/min/1.73m^2^ [14-122] albeit with a relatively short median follow-up time (2.05 years [0.04-7]).

Until more data on the use of growth factors such as teduglutide in patients with SBS and kidney transplantation are available, the definitive way to treat recurrent volume depletion and enteric hyperoxaluria post kidney transplant would be dual kidney and intestinal transplant, or intestine-after-kidney transplant [13, 14]. However, only select centers in the US perform few intestinal transplants yearly, and it could be associated with significant complications [15]. As such, informing patients with SBS and their providers of the high complication rates post kidney transplantation and the potential need for therapies such as regular IVF administration is of paramount importance.

In summary, we describe a favorable outcome of kidney transplantation in a patient with SBS complicated by recurrent AKI treated with at-home IVFs. We also outline for the first time the use of the growth factor teduglutide for SBS after kidney transplant with good result and tolerability albeit with a short follow-up time. We reviewed the available data from similar reported cases which show acceptable posttransplant survival rates and graft function. This case report's major limitations are the descriptive/retrospective nature which is subject to publication bias as well as the relatively short follow-up time. A more systematic and multicenter effort to create a national registry is needed to allow solid conclusions pertinent to the likely advantages of kidney transplantation in patients with SBS.

## Figures and Tables

**Table 1:** Demographics, survival, and graft function in kidney transplant recipients with short bowel syndrome (including the current report).

Number of transplants	20
Age (years)–median (range)	53 (18-74)
Male sex–no. (%)	13 (65)
Cause of SBS–no. (%)	
Crohn's	15 (75)
Obstruction	2 (10)
Other	3 (15)
Cause of ESKD–no. (%)	
Oxalate nephropathy	8 (40)
Nephrolithiasis	4 (20)
Glomerulosclerosis	3 (15)
Other	5 (25)
Follow-up (years)–median (range)	2.05 (0.04-7)
Functioning graft–no. (%)	18 (90)
Death–no. (%)	1 (5)
eGFR (ml/min) at last follow-up–median (range)	37.5 (14-122)
Immunosuppression–no. (%)	
MMF, CNI, and steroids	10 (50)
Azathioprine	3 (15)
Sirolimus	2 (10)
Unknown	4 (20)
Steroid free	1 (5)
Complications–no. (%)	
None	5 (25)
Rejection	3 (15)
Acute allograft dysfunction	12 (60)
Oxalate deposition	5 (25)
Infections	3 (15)

Abbreviations: CNI: calcineurin inhibitors: eGFR: estimated glomerular filtration rate; ESKD: end-stage kidney disease; MMF: mycophenolate mofetil; SBS: short bowel syndrome. Of note, the cumulative number of complications exceeds the number of transplant cases as some patients developed more than one complication.

**Table 2:** Detailed demographics and outcomes of kidney transplants in patients with SBS.

Case	Age	Sex	Cause of SBS	Cause of kidney disease	Transplant	Immunosuppression	Follow-up (years)	eGFR^∗^ (ml/min)	Complications
1 [16]	64	M	Mesenteric thrombosis	Oxalate nephropathy	DDKT	Pred, Aza	0.83	55	Acute rejection
2 [17]	62	F	Intestinal obstruction	Oxalate nephropathy	DDKT	Pred, Aza	7	80	Cryptococcus neoformans infection
3 [18]	74	M	Bowel resection (Crohn's)	Oxalate nephropathy	Unknown	Unknown	3	36	Oxalate deposition
4 [19]	27	M	Ileocolic resection	Congenital urological	DDKT	Pred, MMF, Rapa	1	122	None
5 [20]	40	M	Bowel resection (Crohn's)	Oxalate nephropathy	DDKT	Pred, MMF, Tacro, Rapa	2	14	DGF, oxalate deposition
6 [6]	18	M	Necrotizing enterocolitis	Focal GS	LDKT	M-Pred, MMF, CsA	5	22	Acute rejection, dehydration, acidosis
7 [14]	50	F	Obstruction, EC fistulae	Tacrolimus toxicity	LDKT	Pred, MMF, Tacro	2.1	38	None
8 [2]	38	F	Bowel resection (Crohn's)	Nephrolithiasis	LDKT	Unknown	7	0	Oxalate deposition, IFTA
9 [2]	44	F	Bowel resection (Crohn's)	Oxalate nephropathy	LDKT	MMF, Tacro	1	61	None
10 [2]	49	M	Bowel resection (Crohn's)	Oxalate nephropathy	DDKT	Unknown	3	71	None
11 [2]	47	F	Bowel resection (Crohn's)	Nephrolithiasis	DDKT	Unknown	2	19	Oxalate deposition, GS, tubular atrophy
12 [5]	66	M	Bowel resection (Crohn's)	Oxalate nephropathy	LDKT	Pred, MMF, Tacro	2.6	38	Sepsis at 2 months
13 [5]	63	M	Bowel resection (Crohn's)	Nephrolithiasis	LDKT	Pred, MMF, Tacro	2.58	36	Chronic antibody-mediated rejection
14 [5]	63	M	Bowel resection (Crohn's)	Oxalate nephropathy	DDKT	Pred, MMF, Tacro	2.25	31	Recurrent urinary tract infections
15 [5]	60	F	Bowel resection (Crohn's)	Nephrosclerosis	DDKT	Pred, MMF, Tacro	1.16	99	Delayed graft function, TMA
16 [5]	53	F	Bowel resection (Crohn's)	Unknown	DDKT	Pred, MMF, Tacro	1.16	37	Dehydration, ATN
17 [5]	67	M	Bowel resection (Crohn's)	Nephrolithiasis	DDKT	Pred, MMF, Tacro	1	35	MI, hydro, recurrent UTIs, ATN
18 [5]	72	M	Bowel resection (Crohn's)	Unknown	DDKT	Pred, MMF, Tacro	0.25	69	None
19 [5]	53	M	Bowel resection (Crohn's)	Focal GS	LDKT	Pred, MMF, Tacro	0.04	0	Sepsis, ATN, multiorgan failure, & death
Current	59	M	Bowel resection (Crohn's)	Unknown	DDKT	Pred, Aza, Tacro	2.6	67	DGF, ATN, oxalate deposition

^∗^eGFR at last follow-up was generated using CKD-EPI formula when serum creatinine was available; Caucasian race was assumed if race was not available. ATN: acute tubular necrosis; Aza: azathioprine; DDKT: deceased donor kidney transplant; DGF: delayed graft function; EC: enterocutaneous; GS: glomerulosclerosis; Hydro: hydronephrosis; IFTA: interstitial fibrosis and tubular atrophy; LDKT: living donor kidney transplant; MI: myocardial infarction; MMF: mycophenolate mofetil; Pred: prednisone; Rapa: rapamycin; Tacro: tacrolimus; UTI: urinary tract infection.

## Data Availability

Literature review data could be accessed following the references citing the corresponding article.

## Abbreviations

AKI: Acute kidney injury
CKD: Chronic kidney disease
eGFR: Estimated glomerular filtration rate
GLP-2: Glucagon-like peptide 2
ESKD: End-stage kidney disease
IBD: Inflammatory bowel disease
IVFs: Intravenous fluids
SBS: Short bowel syndrome
sCr: Serum creatinine.
